# Analogues of Y27632 increase gap junction communication and suppress the formation of transformed NIH3T3 colonies

**DOI:** 10.1038/sj.bjc.6605208

**Published:** 2009-08-25

**Authors:** L Hampson, X T He, A W Oliver, J A Hadfield, T Kemp, J Butler, A McGown, H C Kitchener, I N Hampson

**Affiliations:** 1 University of Manchester School of Cancer Studies and Imaging Science, Gynaecological Oncology Laboratories, St Mary's Hospital, Hathersage Road, Manchester M13 OJH, UK; 2 Centre for Molecular Drug Design, Kidscan Laboratories, Cockcroft Building, University of Salford, Manchester M5 4WT, UK

**Keywords:** Y27632, NIH3T3, gap junctions, GJIC, kinase inhibitors, chemoprevention

## Abstract

**Background:**

Constitutive activation of RhoA-dependent RhoA kinase (ROCK) signalling is known to promote cellular transformation and the ROCK inhibitor Y-27632 has the ability to suppress focus formation of RhoA transformed NIH3T3 cells.

**Methods:**

Sixty-four novel structural analogues of Y27632 were synthesised and tested for their ability to persistently inhibit the transformation of NIH3T3 cells by Rho guanidine exchange factor 16 (ARHGEF16) or *Ras*. *In vitro* kinase inhibitor profiling, co-culture of transformed cells with non-transformed cells and a novel Lucifer yellow/PKH67 dye transfer method were used to investigate their mode of action.

**Results:**

Four Y27632 analogues inhibited transformed focus formation that persisted when the compound was withdrawn. No toxicity was observed against either transformed or non-transformed cells and the effect was dependent on co-culture of these two cell types. *In vitro* kinase inhibitor profiling indicated that these compounds had reduced activity against ROCK compared with Y27632, targeting instead Aurora A (AURKA), p38 (MAPK14) and Hgk (MAP4K4). Dye transfer analysis showed they increased gap junction intercellular communication (GJIC) between transformed and non-transformed cells.

**Conclusions:**

These data are the first to suggest that transient blockade of specific kinases can induce a persistent inhibition of non-contact inhibited transformed colony formation and can also remove pre-formed colonies. These effects could potentially be mediated by the observed increase in GJIC between transformed and non-transformed cells. Selection of kinase inhibitors with this property may thus provide a novel strategy for cancer chemoprevention.

The non-transformed murine fibroblast NIH3T3 cell line has been used for many years as a standard assay for the ability of either chemicals and/or targeted alterations of gene expression to induce cellular transformation (Rubin and Xu, 1989). Compared with many other cell systems, malignant conversion of these cells occurs with relative ease. It is usually measured by loss of cell–cell contact growth inhibition, producing colonies or multilayered domes that grow to increased saturation density (Rubin and Xu, 1989). Cells with these properties will typically form tumours when transplanted into nude mice (Rubin, 2005). NIH3T3 cells have been transformed by ectopic expression of many different kinds of gene product and it is particularly relevant to this study that forced expression of proteins, which disturb normal gap junction intercellular communication (GJIC), such as ductin, can transform these cells (Saito *et al*, 1998). Indeed, disruption of GJIC is a common feature of malignant conversion *per se* with the corollary that suppression of transformed cell characteristics can occur when GJIC is established between transformed and non-transformed cells (Yamasaki, 1991; Sakamoto *et al*, 1999).

Earlier studies have also shown that constitutive expression of activated RhoA mutants or guanidine exchange factors can transform NIH3T3 cells and that the formation of transformed colonies can be inhibited by the RhoA kinase (ROCK) inhibitor Y27632 (Sahai *et al*, 1999). Y27632 is a structural analogue of ATP, which has been shown to have potent inhibitory activity against both ROCK's 1 and 2 (Uehata *et al*, 1997; Ishizaki *et al*, 2000). The role of ROCK activation in the process of tumour invasion is well established (Sahai and Marshall, 2002; Croft *et al*, 2004) and there have been several studies on the anti-metastatic potential of Y27632 (Itoh *et al*, 1999; Lawler *et al*, 2006; Ogawa *et al*, 2007). Collectively these data indicate that inappropriate activation of RhoA and thus ROCK proteins may have a role not only in the invasive potential of tumour cells, but also in malignant transformation. A summary of the prospective role of Rho proteins in carcinogenesis is described in Benitah *et al* (2004).

We had shown earlier that interaction of the HPV16 E6 oncoprotein with the PDZ domain protein, Tip-1, may be linked to increased cell motility caused by the activation of RhoA. Furthermore, this increased motility could be inhibited by the ROCK inhibitor Y27632 (Hampson *et al*, 2004). Additional work identified the guanidine exchange factor ARHGEF16 (GEF16) as a novel binding partner for Tip-1 (manuscript in preparation). Thus, when it was observed that transfection of NIH3T3 cells with GEF16 could induce the formation of transformed colonies, we decided to investigate the sensitivity of these transformants to Y27632 and a range of *de novo* synthesised, ATP based, structural Y27632 analogues.

## Materials and methods

### Cell culture and stable gene transfection

The NIH3T3 mouse fibroblast cell line was cultured in DMEM containing 10% bovine serum supplemented with 2 mM L-glutamine and grown at 37°C in humidified air containing 5% CO_2_. The full-length GEF16 open reading frame (Accession NM_014448) was PCR amplified, sequence verified and sub-cloned into the mammalian expression vector pCMVTag (Invitrogen Ltd., Paisley, UK). The pCMVTag-GEF16 cDNA construct or the LZR-MS-IRES-ZEO/pBR-*Ras* construct (A kind gift of Dr A Malliri; PICR, Manchester, UK) was then used to transfect NIH3T3 cells using Lipofectamine according to the manufacturer's recommendations (Invitrogen Ltd.). GEF16, *Ras* and vector control transfected cells were then maintained in the presence of G418 or Zeocin for 10 days. Polyclonal GEF16, *Ras* and vector transfectants were expanded in sub-confluent cultures and −80°C freezer stocks taken. Individual GEF16 transformed colonies were isolated by the use of cloning rings, expanded in culture and −80°C frozen stocks also taken for storage.

### RT–PCR

Total cellular RNAs were prepared using the SuperScript III cells direct cDNA synthesis kit as recommended by the manufacturer (Ambion, Cambridgeshire, UK). Total RNAs from cells were isolated using Trizol reagent (Invitrogen Ltd.). All DNAase I-treated RNAs were then reverse transcribed with random decamers. Polymerase chain reaction was performed in 20 *μ*l of a reaction mixture containing 2 *μ*l of reverse-transcribed product, 10 *μ*l of 2 × Bio-Red (bioline Ltd., London, UK) and 0.1 *μ*M of each primer. The specific primers for GEF16 and *β*-actin were as follows:

GEF16 forward: 5-ACCACCACCTCTTCTCCAAC-3′

GEF16 reverse: 5′-TCGTTGGAGCAGTAGGCGAT-3′

*β*-actin forward: 5′-TCC ATC ATG AAG TGT GAC GT-3′

*β*-actin reverse: 5′-TCA GGA GGA GCA ATG ATC TT-3′

The reaction mixture was denatured at 94°C for 4 min then amplified for 32 cycles of 30 s denaturation at 94°C, 30 s annealing at 55°C and 30 s extension at 72°C, followed by a single 5 min extension at 72°C.

### Transformed colony forming assay

Polyclonal vector or GEF16 transfected NIH3T3 cells were seeded separately in 30 mm dishes at a density of 2 × 10^5^ cells per well and grown to full confluence in the presence of either 10 *μ*M Y27632 (Calbiochem, Darmstadt, Germany), Y27632 analogues (YA1, YA2, YA3, YA4) or DMSO control. Medium plus inhibitors or DMSO control was changed every 2 days and foci formation analysed by Toluidine blue (Sigma-Aldrich, Poole, UK) staining after 10 days growth post-confluence. Each assay was performed in triplicate and the data shown is representative of at least three separate experiments.

### *In vitro* ROCK activity assay

Rho-kinase activity was determined using an immunoassay as recommended by the manufacturer (CycLex Co., Ltd., Nagano, Japan). Briefly, 100 *μ*l samples containing 10 mUnit of recombinant ROCK with or without inhibitors were aliquoted into a 96-well plate, pre-coated with threonine Rho-kinase phosphorylation substrate. After 30 min incubation at 30°C, the plate was washed three times with PBS then incubated with 100 *μ*l/well of HRP-conjugated anti-phospho-specific antibody for 1 h at room temperature. The amount of phosphorylated substrate was determined by adding 100 *μ*l/well of substrate reagent for 10 min and the reaction was terminated by adding 100 *μ*l/well of the stop solution. The absorbance was measured on a 96-well plate reader at 450 nm (Dynex Technologies, West Sussex, UK). Each data point was performed in triplicate and the assay was repeated twice.

### SelectScreen *in vitro* kinase profiling

The SelectScreen kinase inhibitor assay service was used (Invitrogen Ltd.). The YA compounds were diluted in DMSO at a concentration of 10 mM and single-point kinase inhibitory activities were measured at 10 *μ*M and Km ATP concentration.

### Cell proliferation assay

Cell proliferation was measured Celltiter Aq^96^ reagent (Promega, Southhampton, UK) according to the manufacturer's protocol. Cells were seeded into a 96-well plate at a density of 1 × 10^3^ cells/well allowing three wells per data point and allowed to attach for a set period. After this, the initial starting point 490 nm absorbance was determined by adding 20 *μ*l of Aq^96^ reagent to each well and incubating for 4 h at 37°C in 5% CO_2_ (96-well plate reader, Dynex Technologies). The various compounds or DMSO control were then added to the wells and the absorbance determined in the same way at the time points indicated. Each data set shown is representative of three separate experiments.

### Flow cytometry

NIH3T3 cells with or without drug treatments were collected at various time points and cell counts carried out to confirm that 1 × 10^6^ cells were present for each cytometric analysis. Cells were washed with PBS, fixed with 70% ice-cold ethanol, pelleted and stained with propidium iodide (10 mg ml^−1^) (Sigma-Aldrich) at 4°C for 45 min. Cells were then washed twice with ice-cold PBS and resuspended in 400 *μ*l of PBS. The DNA content during different phases of the cell cycle was then determined by flow cytometry FACSConto (BD Biosciences, Oxford, UK). Each profile shown was a representative of three separate experiments.

### Quantitative analysis of GJIC

A flow cytometric assay was developed to measure the extent of GJIC using two differentially stained cell populations. Non-transformed recipient NIH3T3-vector cells were stained with PKH67 (excitation 490, emission 502, Sigma-Aldrich) and GEF16 transformed donor cells were stained with Lucifer yellow (LY) (excitation 427 nm, emission 517 nm, Invitrogen Ltd.). A 1 ml suspension of 1 × 10^7^ recipient cells in serum-free DMEM was mixed with an equal volume of 4 *μ*M PKH67 solution and incubated for 5 min at room temperature. The reaction was terminated by adding 2 ml serum and incubating for 1 min. Cells were then washed three times with culture medium, and seeded at 1.5 × 10^6^ cells/T-25 flask. For LY staining of transformed donor cells, 700 *μ*l of 5 × 10^6^ cells were mixed with 100 *μ*l of 8 mg ml^−1^ LY solution in a 4 mm gap electroporation cuvette (EquiBIO, Middlesex, UK) and this was kept on ice for 5 min followed by electroporation at 400 V (1000 V cm^−1^) (Gene Transformer, Savant Instruments Inc., NY, USA). Fresh medium was added and the cells were seeded in a T-25 flask and were allowed to recover overnight at 37°C. Lucifer yellow-labelled donor cells were then collected and 1 × 10^5^ were added to the T-25 flask containing the PKH67-labelled recipient cells plus 10 *μ*M YA1 or DMSO control. After incubating for various time intervals, the co-cultured donor and recipient cells were collected and analysed using a BD FACS Aria (GJIC, BD Biosciences). A 405 nm laser was used for LY excitation and emission was measured using a 515–545 nm band pass filter. Gap junction intercellular communication between co-cultured donor and recipient cells was quantified as the percentage of LY and PKH67 double-labelled cells.

### Statistical analysis

All data presented are from single or paired experiments performed in triplicate, or from 2–3 separate experiments in duplicate. Comparisons between groups were performed using paired or un-paired two-tailed Student's *t*-test. Statistical significance was taken to represent a *P*-value <0.05.

## Results

### Constitutive expression of GEF16 transforms NIH3T3 cells

**Figure 1:**
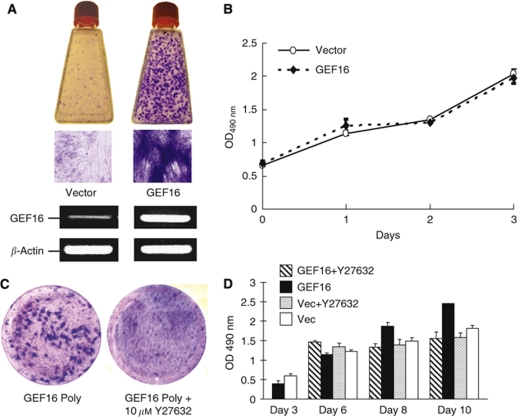
The ROCK inhibitor Y27632 suppresses the formation of GEF16 transformed NIH3T3 colonies. (**A**) NIH3T3 fibroblasts were transformed by stable transfection with GEF16 cDNA followed by 12 days of growth in the presence of G418. Transformed foci were visualised by Toluidine blue staining and GEF16 mRNA expression was verified by RT–PCR. (**B**) Cell AQ^96^ growth comparison of vector and GEF16 polyclonal transfected cells (**C**) Polyclonal GEF16 transfected NIH3T3 cells were incubated with either 10 *μ*M of Y27632 or DMSO control for 10 days and stained with Toluidine blue. (**D**) GEF16 and vector transfected cells were seeded in a 96-well plate at 1 × 10^3^ cells per well. These were incubated for 3 days followed by addition of Cell AQ^96^ reagent to determine the starting point for the assay. A measure of 10 *μ*M of either Y27632 or DMSO was then added to wells containing both cell types and Cell AQ^96^ absorbance measured at 6, 8 and 10 days. At day 6 cells were confluent, which was determined by phase contrast visual inspection of the cultures at × 20 magnification.

As discussed earlier NIH3T3 cells can be transformed by either ectopic expression of constitutively activated RhoA (Sahai *et al*, 1999) or various other guanidine exchange factors (Brecht *et al*, 2005). Our results are consistent with these data because they clearly show that constitutive expression of GEF16 mRNA induces the formation of multiple transformed foci in NIH3T3 cells after 12 days of growth in the presence of G418 (Figure 1A). Multilayered transformed G418-resistant colonies were picked for further analysis and no transformed foci were observed in G418 selected vector transfected control cells. Comparison of the growth of vector and GEF16 transfected cells shows that there is no significant difference in proliferation rates between these two cell types (Figure 1B; *P*>0.05).

### Y27632 inhibits the formation of GEF16 transformed colonies

Consistent with the work of Sahai *et al* (1999) our results show that treatment with 10 *μ*M of the ROCK inhibitor, Y27632, for 10 days suppresses the formation of GEF16 transformed NIH3T3 cell colonies (Figure 1C). However, it can also be seen that Y27632 inhibits the growth of confluent GEF16 transformed cells, yet these cells continue to proliferate in identical untreated cultures (Figure 1D).

### Structural analogues of Y27632 inhibit the growth of GEF16 transformed colonies

**Figure 2:**
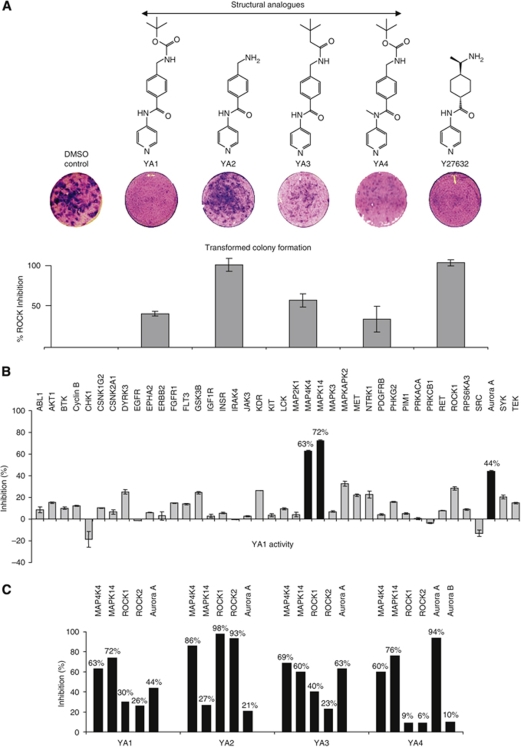
Structural analogues of Y27632 also inhibited the formation of GEF16 transformed colonies but some had reduced ROCK inhibitory activity. (**A**) Aliquots of 2.0 × 10^5^ polyclonal GEF16 transfected cells were seeded into 30 mm dishes, incubated overnight then treated with 10 *μ*M of 64 different structural analogues of Y27632. Transformed colony formation was assayed by Toluidine blue staining after 10 days. The ROCK inhibitory activity of four of these compounds (YAs 1–4) with biological activity was compared with Y27632 (**B**) The kinase inhibitory activity of YA1 was initially evaluated against a representative selection of 40 human kinases (SelectScreen). (**C**) Single-point analysis of the kinase inhibitory activity of YAs 1–4 against HGK, p38, ROCKs 1 and 2 and Aurora A.

Y27632 is a structural analogue of ATP and 64 different analogues of this inhibitor were synthesised with the intention of evaluating their ability to inhibit the formation of GEF16 transformed NIH3T3 foci. At 10 *μ*M, none of the 64 compounds showed any appreciable growth inhibitory activity (data not shown), yet four had the ability to suppress the formation of GEF16 transformed colonies growing in post-confluent cultures (Figure 2A). The structures of these are shown with YA1 being the most potent and YA2 the least. Using this simple assay, the ability of YA1 to block transformed GEF16 colony formation is comparable to that of Y27632.

### YA1, YA3 and YA4 have reduced ROCK inhibitory activity whereas YA2 has equivalent activity to Y27632

*In vitro* assays of inhibitory activity against ROCK indicated that YA1, YA3 and YA4 had significantly less activity against this kinase than Y27632 (Figure 2A). However, paradoxically YA2, which was least effective at preventing GEF16 colony formation, had comparable ROCK inhibitory activity to Y27632. On the basis of these results it was concluded that the ability of compounds YA1, YA3 and YA4 to inhibit GEF16 colony formation may be either because of inhibitory effects against unidentified kinases or other alternative cellular targets.

### YA1, YA3 and YA4 have inhibitory activity against p38, HGK and Aurora A kinases whereas YA2 targets HGK and ROCKs 1 and 2

As YA1, YA3 and YA4 are structurally very similar and YA1 was the most potent at blocking GEF16 transformed colonies (Figure 2A), an *in vitro* kinase inhibitory assay was performed on this compound against a representative selection of 40 human kinases (SelectScreen) (Figure 2B). These data show that at 10 *μ*M, YA1 had maximal inhibitory activity against p38 *α* (MAPK14) (72%), HGK (MAP4K4) (63%) and Aurora A (44%) and also confirmed the reduced ROCK inhibitory activity of YA1 shown in Figure 2A (∼40%). An additional single-point analysis of the inhibitory activity of 10 *μ*M of compounds YA's 1, 2, 3 and 4 against p38, HGK, Aurora A, and ROCKs 1 and 2 confirmed that YA2 had the greatest activity against ROCK's 1 and 2. YAs1, 3 and 4 all have significant activity against p38, HGK and Aurora A, but show less activity against ROCKs than YA2. Interestingly, YA2 also has the greatest inhibitory activity against HGK (Figure 2C) but is the least effective at preventing colony formation (Figure 2A). Paradoxically, even though YA1 is the most potent in cell-based assays, it has roughly equivalent activity to YA4 against HGK and p38 yet has less inhibitory activity against Aurora A than YA4. These observations suggest that, although the three kinases identified as targets of YA1 cannot be excluded as having a role in the formation of GEF16 transformed colonies, it is clear that this compound may also affect other, as yet, unidentified cellular targets.

### Transient exposure of GEF16 cells to either YA1 or Y27632 eliminates transformed colony forming cells from polyclonal GEF16 cells

**Figure 3:**
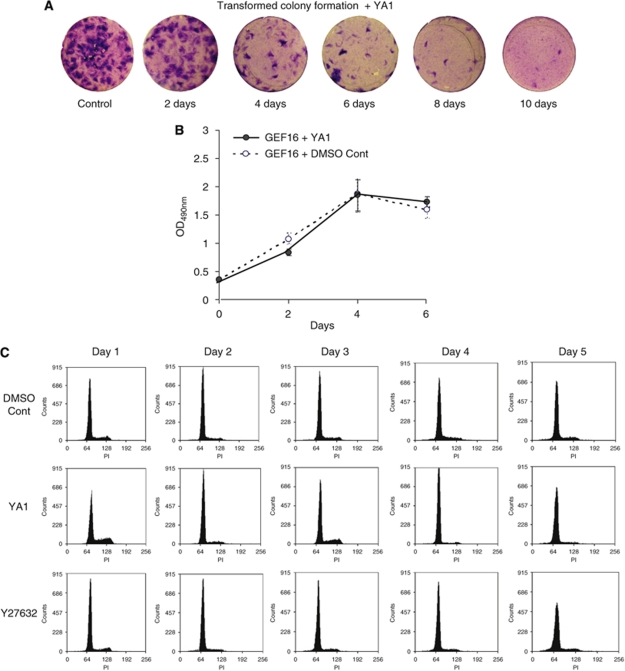
YA1 irreversibly suppresses the formation of GEF16 transformed NIH3T3 colonies. (**A**) Polyclonal GEF16 transfected cells were plated at 2.0 × 10^5^ cells per 30 mm dish and 10 *μ*M of YA1 added for 2, 4, 6, 8 and 10 days, respectively. After this incubation period the compound was then removed from the culture media and cells maintained in normal growth media for a further 10 days. (**B**) Cell AQ^96^ proliferation assay of sub-confluent cultures treated with DMSO or inhibitor for the same time interval. (**C**) Flow cytometric analysis of either YA1 or Y27632 (10 *μ*M)-treated GEF16 cells.

**Figure 4:**
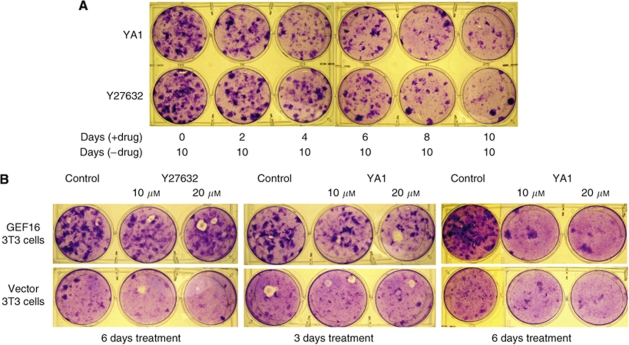
YA1 and Y27632 irreversibly suppress the formation of GEF16 transformed NIH3T3 colonies whereas, unlike Y27632, YA1 eliminates pre-existing transformed colonies. (**A**) Polyclonal GEF16 transfected cells were seeded at 2.0 × 10^5^ cells per 30 mm dish and incubated overnight. A measure of 10 *μ*M YA1 or Y27632 was then added to each of these for 2, 4, 6, 8 and 10 days, respectively, where upon the cells were detached with trypsin and re-plated at a density of 2.0 × 10^5^ cells. After a further 10 days culture in the absence of inhibitors the cells were stained with Toluidine blue. (**B**) Polyclonal GEF16 and vector transfected cells were seeded at 2.0 × 10^5^ cells per 30 mm dish and incubated for 10 days after which 10 or 20 *μ*M of either, YA1, Y27632 or DMSO control was added. These were incubated for 3 or 6 days then stained with Toluidine blue.

Freshly plated GEF16 polyclonal cells were treated with 10 *μ*M of YA1 for 2, 4, 6, 8 and 10 days, respectively, after which the compound was removed from the culture media and the cells maintained in normal media for a further 10-day chase period. This shows a progressive decrease in the number of transformed foci associated with increased time of exposure to the compound (Figure 3A) and indicates that YA1 not only inhibits the formation of transformed foci but, on withdrawal, also prevents transformed foci from reforming. Significantly, there is no detectable difference in growth rates of sub-confluent GEF16 polyclonal cells treated with either inhibitor YA1 or DMSO control (Figure 3B) (*P*>0.05), and flow cytometry shows no evidence of alterations in cell cycle or the accumulation of an apoptotic sub-G1 population (Figure 3C). In Figure 3A the number of cells per dish increases with each successive inhibitor-treatment time interval before withdrawal of the compound. To remove this variable and to ensure that each 10-day chase period starts with the same number of inhibitor-treated cells, GEF16 polyclonal cells were treated with either inhibitor YA1 or Y27632 for 2, 4, 6, 8 and 10 days. After this, cells were detached, re-seeded at 2 × 10^5^ per well in 6-well plates and then maintained in the absence of inhibitors for a 10-day chase period. This shows suppression of transformed focus formation by either YA1 or Y27632 treatment, which correlates with the duration of exposure (Figure 4A). Collectively these observations are the first to show that transient treatment with Y27632 or YA1 permanently suppresses transformed colony formation and does not involve cell killing.

### YA1 eliminates pre-formed transformed colonies from GEF16 polyclonal cells

The results have so far shown that both Y27632 and YA1 can prevent transformed colonies from forming and that this effect persists. To evaluate the effects of these compounds on pre-formed colonies, polyclonal GEF16 NIH3T3 transformed colonies were allowed to form for 10 days and then exposed to Y27632 or YA1 for 3 and 6 days. It can be seen that Y27632 has very little effect on pre-formed transformed colonies whereas YA1 causes a marked reduction in their numbers (Figure 4B). Vector transfected polyclonal NIH3T3 cells are included as a control and show no difference between inhibitor treated and DMSO controls.

### YA1 has minimal effects on cells derived from single GEF16 transformed colonies

**Figure 5:**
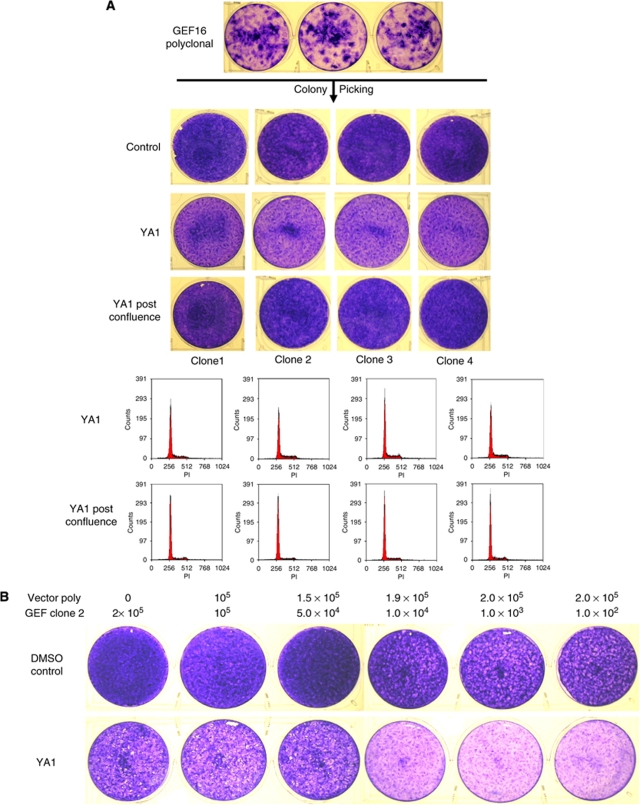

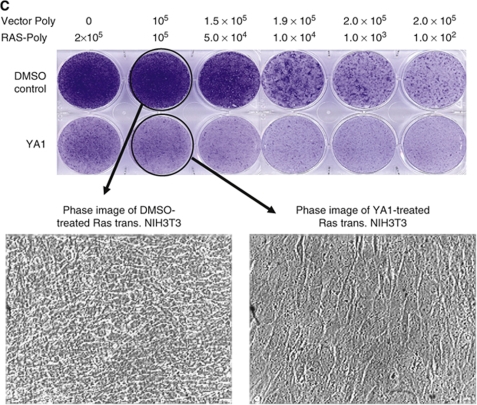
The growth suppressive effects of YA1 on single transformed colony-derived GEF16 NIH3T3 cells and polyclonal *Ras* transformed NIH3T3 cells are more pronounced when these are co-cultured with non-transformed cells. (**A**) Single transformed colonies were picked from 10-day cultures of GEF16 polyclonal NIH3T3 cells and expanded. These cells were then seeded at 2.0 × 10^5^ cells per 30 mm dish and treated with 10 *μ*M of YA1 or DMSO control either immediately or after 10 days in culture. Duplicate wells were collected for flow cytometry. (**B**) A total of 2 × 10^5^ cells per well were plated consisting of increasing numbers of non-transformed vector cells co-cultured with decreasing numbers of single transformed colony-derived GEF16 cells. These were treated with 10 *μ*M of either YA1 or DMSO for 10 days. (**C**) The same co-culture experiment described in (**B**) was performed substituting polyclonal *Ras* transformed NIH3T3 cells for GEF16 transformed cells.

Single transformed colonies were picked from GEF16 polyclonal NIH3T3 cells and expanded (Figure 5A). These were plated and inhibitor YA1 added either immediately or after 10 days when the cells were post-confluent. It can be seen that this has a modest effect when added to low density cultures whereby these do not achieve the same saturation density as control cells. However, addition of YA1 to post-confluent cultures has no discernible effect when compared with controls. Flow cytometry shows YA1 has no effect on the cell cycle and no apoptotic sub-G1 population is seen in cultures treated either pre- or post-confluence.

### YA1 suppresses the growth of monoclonal GEF16 and polyclonal *Ras* transformed cells when these are co-cultured with non-transformed NIH3T3 cells

Non-transformed vector cells mixed with decreasing numbers of cells expanded from single GEF16 transformed colonies were treated with inhibitor YA1 or DMSO and incubated for 10 days. It can be seen that YA1 causes a marked reduction in the final saturation density of the cultures and that this is dependent on the number of transformed cells plated (Figure 5B). Comparing these results to those shown in Figure 5A indicates that YA1 is more effective at suppressing the growth of transformed cells when these are in contact with non-transformed cells. Figure 5C shows that YA1 produces the same growth suppressive effects on *Ras* transformed NIH3T3 cell colonies as with GEF16 transformed cells and that these are also dependent on contact with non-transformed cells. The phase contrast images also show how YA1-treated *Ras* transformed cells regain both contact inhibition and polarity.

### YA1 increases intercellular GJIC between transformed and non-transformed cells

**Figure 6:**
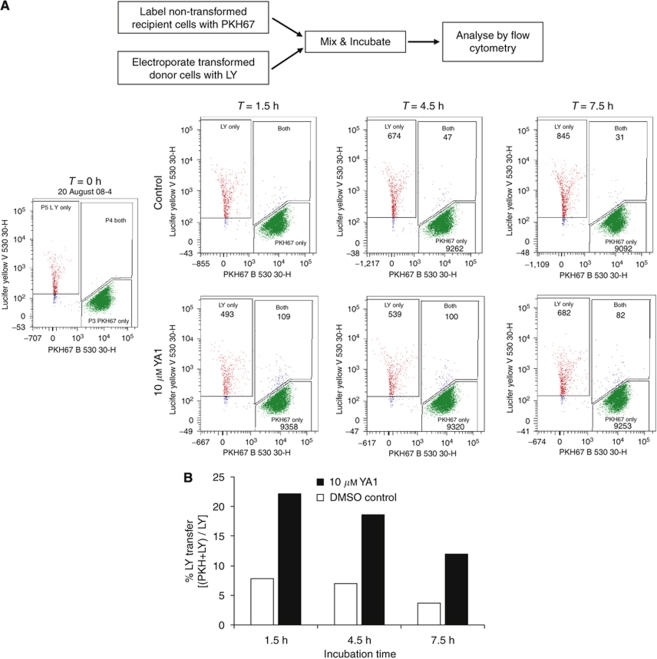
YA1 stimulates GJIC between transformed and non-transformed cells. (**A**) Cells derived from a single GEF16 transformed colony were electroporated with LY and co-cultured with non-transformed cells that had been labelled earlier with PKH67. Either YA1 (10 *μ*M) or DMSO control was added to duplicate cultures and these were collected at *T*=0, 1.5, 4.5 and 7.5 h for analysis by flow cytometry. The numbers of cells displaying each type of fluorescence is shown. (**B**) The ratio of double LY/PKH67-labelled cells expressed as percentage of the total LY population.

Figure 6A shows the effect of either YA1 or DMSO control on the extent of dye transfer from co-cultured donor LY-labelled GEF16 single colony transformed cells to recipient PKH67 stained non-transformed cells. It can be seen from Figure 6B that YA1-treated cultures have approximately three times the number of double LY/PKH67-labelled cells when compared with DMSO control (*P*<0.05). This indicates an increase in the transfer of dye from LY to PKH67 stained cells, which is consistent with an inhibitor-induced increase in GJIC.

## Discussion

Our data are the first to show that transient treatment with either the ROCK inhibitor, Y27632, or specific structural analogues of this compound not only prevents the formation of transformed NIH3T3 colonies, but also colonies do not form when the compounds are withdrawn. Most surprising was that, unlike Y27632, the effects of YA1, 3 and 4, occur independent of ROCK inhibitory activity and do not involve cell killing. Furthermore, YA1 has the additional property of eliminating pre-existing transformed colonies and this effect is also not produced by cell killing.

In an attempt to improve the potency of Y27632, 64 different structural analogues were synthesised of which 3 were initially shown to have similar effects to Y27632 on transformed colony formation, yet these compounds had lower ROCK inhibitory activity. Paradoxically, although YA2 had equivalent ROCK inhibitory activity to Y27632, it was much less effective at preventing transformed colony formation. Clearly this could be due to the pharmacodynamic propertes of this compound although an alternative explanation could be that the effects of Y27632 are not entirely because of ROCK inhibition. For example, in addition to ROCK's 1 and 2 it is known that at 10 *μ*M Y27632 also has significant inhibitory activity against 16 other kinases including the protein kinase C (PKC) isoforms *β*, *ε* and *η*, and the myotonic dystrophy kinase-related Cdc42-binding kinases Cdc42 BPA (MRCKA) and Cdc42 BPB (MRCKB). (For a complete list of these see supplementary information URL: http://www.invitrogen.com/etc/medialib/en/filelibrary/pdf.Par.96408.File.dat/SelectScreen_Data_193.pdf). The Cdc42-activated MRCK kinases are particularly relevant because, similar to ROCKs, they promote myosin-dependent cell motility and indicate a point of convergence between RhoA and Cdc42 signalling (Wilkinson *et al*, 2005). Thus, the observed inability of compound YA2 to prevent the formation of transformed colonies could be due to its lack of inhibitory activity against one or more of these alternative target kinases. Indeed, PKC epsilon undergoes 94% inhibition by 10 *μ*M Y27632 and it has been shown that increased activity of this kinase is causally associated with calpain inhibitor-induced transformation of NIH3T3 cells (Hiwasa *et al*, 2002). In addition, GEF16 possesses a potential Cdc42-binding motif (Blanke and Jackle, 2006) and we have shown that GEF16 specifically activates Cdc42 *in vitro* and in cells (See Supplementary Data Figure 1; manuscript in preparation). Interestingly, activated Cdc42 is also known to promote the activation of p38 (Hall, 2005), which is entirely consistent with the p38 inhibitory activity of compounds YA1, YA3 and YA4.

However, it is clear from Figure 2 that there is not an obvious relationship linking any of the kinases identified as being the sole target responsible for the biological activity of YA1. This is either the result of inhibition of an, as yet, unidentified kinase or may reflect a particular combination of inhibitory activities.

The observed minimal toxicity combined with persistence of the suppressive effects of the YA compounds on transformed colony formation, prompted an investigation into the rationale behind this effect. As discussed earlier, the ability of non-transformed cells to establish GJIC with transformed cells has been shown to suppress the transformed properties of cells without cell killing (Sakamoto *et al*, 1999). Thus, we hypothesised that inhibitor induced increased GJIC between transformed and non-transformed NIH3T3 cells is the most plausible mode of action (Sakamoto *et al*, 1999). To address this question we developed the LY/PKH67 vital dye staining method. Lucifer yellow cannot penetrate cell membranes (Raptis *et al*, 1994) and PKH67 remains very stably associated with labelled cells (Rousselle *et al*, 2001). Our method clearly shows that YA1 induces increased accumulation of double LY/PKH67-labelled cells when compared with DMSO-treated controls, which strongly supports a YA1-mediated increase in GJIC between transformed and non-transformed cells.

If this rationale is correct, treatment of single colony-derived GEF16 transformed NIH3T3 cells with either YA1 or Y27632 should have little or no effect when these are grown in the absence of non-transformed cells and results shown in Figure 5A support this. However, the growth inhibitory effects of YA1 on single colony-derived transformed cells are clearly restored when these are co-cultured with non-transformed NIH3T3 cells (Figure 5B). A surprising and unexpected result was the ability of this compound to eliminate pre-formed transformed colonies from GEF16 polyclonal cells (Figure 5). Neither Y27632 nor any of the other compounds tested (data not shown) had this property, which clearly distinguishes YA1 from all the others and suggests an additional mode of action.

Is there any evidence that the various kinase targets described may participate in transformation-induced disruption of gap junctions? Inhibition of ROCK activation by Y27632 has been shown to facilitate the formation of gap junctions in corneal epithelium (Anderson *et al*, 2002). Furthermore, *H-Ras*-induced disruption of gap junctions in rat liver epithelial cells can be reversed by treatment with the p38 inhibitor SB203580 (Lee *et al*, 2004). We found that SB203580 had some activity against GEF16 transformed colonies although this was much less than YA1 (see Supplementary Data Figure 2). Considering that 10 *μ*M SB203580 has >20-fold more p38 inhibitory activity than YA1 at the same concentration (Tocris Biosciences, Bristol, UK; Data Sheet) this implies that p38 inhibition alone does not explain the inhibitory activity of YA1 against transformed colonies.

It is very significant that YA1 was also highly effective at preventing the formation of *Ras* transformed NIH3T3 colonies, which indicates that the activity of these compounds is not restricted to a specific GEF but instead targets Rho/Ras-mediated transformation in general.

In summary, our data support the hypothesis that specific inhibitors with the ability to modulate the activity of selected kinases may form the basis of a novel strategy for cancer chemo-prevention. The effect is most likely produced by enhancement of the ability of non-transformed cells to establish GJIC with transformed cells and it can clearly persist after withdrawal of the inhibitor. Most importantly, if these findings can be translated into an *in vivo* cancer chemoprevention strategy, it suggests that the active agent may not need to be administered continuously. Furthermore, we would also suggest that inhibitors of this type should be explored as a means of suppressing both the formation and growth of metastases. It is highly significant that Y27632 has been shown to suppress the development of metastases *in vivo* (Itoh *et al*, 1999; Ogawa *et al*, 2007) with the rationale being that ROCK inhibition suppresses the migration of tumour cells. Our data indicate that if Y27632 or our novel compounds can promote intercellular communication between metastatic cells and normal cells at distant sites of invasion, this could provide an additional unexplored level of growth control during the metastatic process and we are currently exploring this hypothesis.
