# Supplementary material for: Analogues of Y27632 increase gap junction communication and suppress the formation of transformed NIH3T3 colonies
Source: Br J Cancer. 2009 Aug 25;101(5):829–39. doi: 10.1038/sj.bjc.6605208 (PMC2736836; doi:10.1038/sj.bjc.6605208)
Supplement: Supplementary Material [file 6605208x1.pdf]

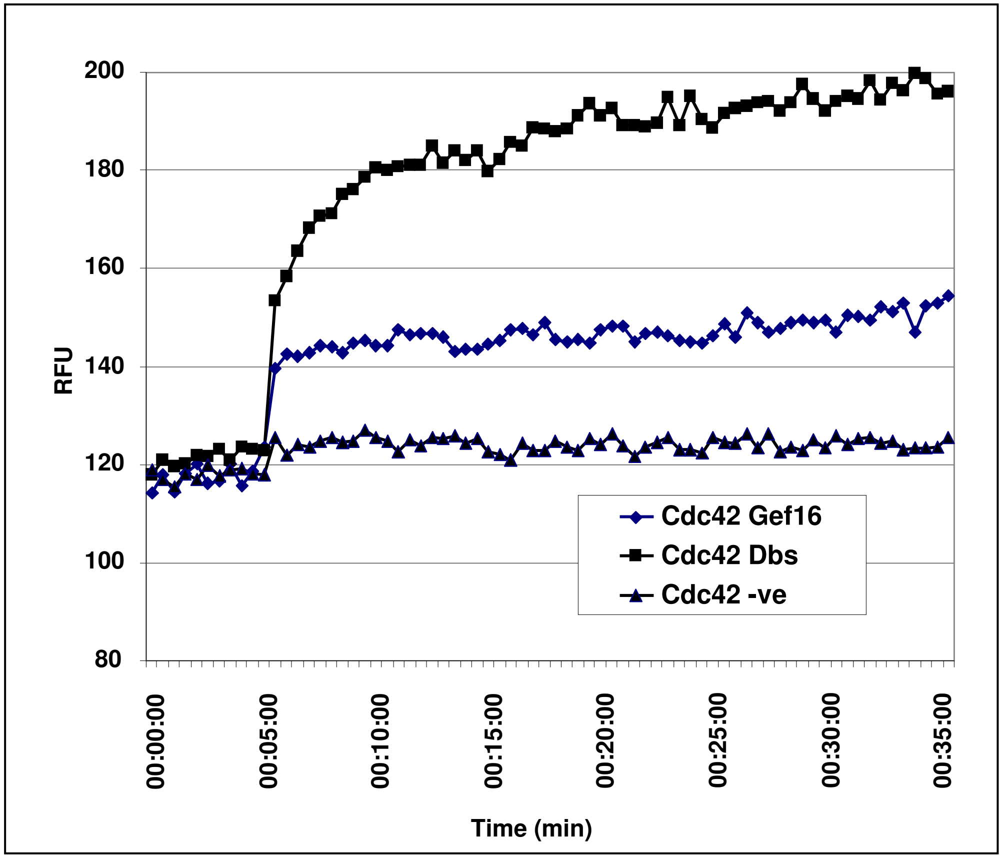

Supplementary Figure 1. Fluorescent kinetic analysis showing the ability of recombinant GEF16 to activate Cdc42 in vitro. Recombinant GEF16 (GenWay Biotech, San Diego, CA, USA) was used in the RhoGEF exchange assay kit (Cytoskeleton, Denver, CO, USA). 2 uM concentrations of GEF16 were assayed against RhoA, Rac and Cdc42 according to the manufacturer's instructions using a Spectra Max Gemini XS Microplate Spectrofluorometer (Molecular Devices Sunnyvale, CA, USA). Addition of recombinant GEF16 protein to Cdc42 produces a marked increase in the transfer and exchange of GTP for GDP bound to Cdc42 which is approximately half the rate seen with the Dbs GEF positive control. Using this same assay GEF16 did not cause activation of the other Rho proteins Rac1 or RhoA (Data not shown).

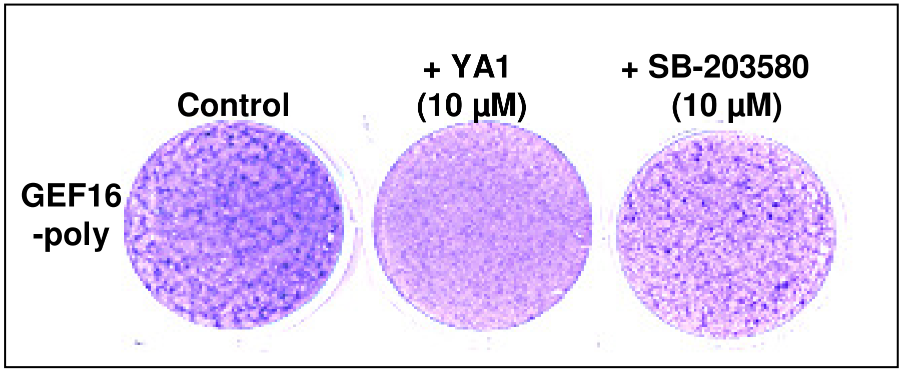

Supplementary Figure 2. p38 inhibitor SB-203580 shows minimal inhibition on the formation of GEF16 transformed NIH3T3 colonies Aliquots of 2.0 x 10^5 polyclonal GEF16 transfected cells were seeded into 30 mm dishes and incubated over night. In order to compare SB-203580 with YA1 in the effect of GEF16 colony formation, cells were then treated with 10 µM of YA1 and SB-203580 respectively. Transformed colony formation was assayed by toluidine blue staining after 10 days. The figure shows the data from 2 separate experiments carried out in duplicate. Even though the cells were exposed to SB-203580 at high dose (>20 folds of IC_{50}), the colony formation was only partially inhibited.
